# Serial Anti–Myelin Oligodendrocyte Glycoprotein Antibody Analyses and Outcomes in Children With Demyelinating Syndromes

**DOI:** 10.1001/jamaneurol.2019.2940

**Published:** 2019-09-23

**Authors:** Patrick Waters, Giulia Fadda, Mark Woodhall, Julia O’Mahony, Robert A. Brown, Denise A. Castro, Giulia Longoni, Sarosh R. Irani, Bo Sun, E. Ann Yeh, Ruth Ann Marrie, Douglas L. Arnold, Brenda Banwell, Amit Bar-Or

**Affiliations:** 1 Nuffield Department of Clinical Neurosciences, John Radcliffe Hospital, University of Oxford, Oxford, United Kingdom; 2 Perelman Center for Advanced Medicine, Department of Neurology, University of Pennsylvania, Philadelphia; 3 Institute of Health Policy, Management and Evaluation, The Hospital for Sick Children, University of Toronto, Toronto, Ontario, Canada; 4 Montreal Neurological Institute, McGill University, Montreal, Quebec, Canada; 5 Department of Diagnostic Imaging, The Hospital for Sick Children, University of Toronto, Toronto, Ontario, Canada; 6 Hospital for Sick Children Research Institute, Division of Neurology, Department of Pediatrics, University of Toronto, Toronto, Ontario, Canada; 7 Department of Internal Medicine, Rady Faculty of Health Sciences, Max Rady College of Medicine, University of Manitoba, Winnipeg, Manitoba, Canada; 8 Department of Community Health Sciences, Rady Faculty of Health Sciences, Max Rady College of Medicine, University of Manitoba, Winnipeg, Manitoba, Canada; 9 Division of Neurology, The Children’s Hospital of Philadelphia, Philadelphia, Pennsylvania; 10 Center for Neuroinflammation and Experimental Therapeutics, University of Pennsylvania, Philadelphia

## Abstract

**Importance:**

Identifying the course of demyelinating disease associated with myelin oligodendrocyte glycoprotein (MOG) autoantibodies is critical to guide appropriate treatment choices.

**Objective:**

To characterize serial anti-MOG antibody serologies and clinical and imaging features at presentation and during follow-up in an inception cohort of prospectively monitored children with acquired demyelination.

**Design, Setting, and Participants:**

In this prospective cohort study, study participants were recruited from July 2004 to February 2017 through the multicenter Canadian Pediatric Demyelinating Disease Study. Inclusion criteria included (1) incident central nervous system demyelination, (2) at least 1 serum sample obtained within 45 days from onset, and (3) complete clinical information. Of 430 participants with acquired demyelinating syndrome recruited, 274 were included in analyses. Of 156 excluded participants, 154 were excluded owing to missing baseline samples and 2 owing to incomplete clinical information. Data were analyzed from May to October 2018.

**Main Outcomes and Measures:**

Presence of anti-MOG antibodies was blindly assessed in serial samples collected over a median of 4 years. Clinical, magnetic resonance imaging, and cerebrospinal fluid features were characterized at presentation, and subsequent disease course was assessed by development of new brain magnetic resonance imaging lesions, total lesion volume at last evaluation, annualized relapse rates, Expanded Disability Status Scale score and visual functional score at 4 years, and any disease-modifying treatment exposure.

**Results:**

Of the 274 included participants, 140 (51.1%) were female, and the median (interquartile range) age of all participants was 10.8 (6.2-13.9) years. One-third of children were positive for anti-MOG antibodies at the time of incident demyelination. Clinical presentations included a combination of optic neuritis, transverse myelitis, and acute disseminated encephalomyelitis for 81 of 84 anti-MOG antibody–positive children (96%). Brain lesions were present in 51 of 76 anti-MOG antibody–positive participants (67%), but magnetic resonance imaging characteristics differed with age at presentation. Complete resolution of baseline lesions was observed in 26 of 49 anti-MOG antibody–positive participants (53%). On serial serum analysis, 38 of 67 participants (57%) who were seropositive at onset became seronegative (median time to conversion, 1 year). Among all participants who were positive for anti-MOG antibodies at presentation, clinical relapses occurred in 9 of 24 children (38%) who remained persistently seropositive and in 5 of 38 children (13%) who converted to seronegative status.

**Conclusions and Relevance:**

Myelin oligodendrocyte glycoprotein antibodies are common in children with acquired demyelinating syndrome and are transient in approximatively half of cases. Even when persistently positive, most anti-MOG antibody–positive children experience a monophasic disease. The presence of anti-MOG antibodies at the time of incident demyelination should not immediately prompt the initiation of long-term immunomodulatory therapy.

## Key Points

**Question:**

Are antibodies to myelin oligodendrocyte glycoprotein (MOG) associated with relapses in children with acquired demyelination?

**Findings:**

In this cohort study including 274 children with acquired demyelinating syndrome, anti-MOG antibodies were found in approximately 30% of children at presentation; they were more frequent in children with acute disseminated encephalomyelitis or younger than 11 years and were rarely present in children meeting multiple sclerosis diagnostic criteria. Neither presence of anti-MOG antibodies at onset nor their subsequent persistence were strongly associated with relapsing disease.

**Meaning:**

While common in children with demyelination, anti**-**MOG antibodies should not be used to adjudicate long-term immunomodulatory therapy in the absence of clinical relapsing disease.

## Teaser

This cohort study characterizes serial anti–myelin oligodendrocyte glycoprotein antibody serologies and clinical and imaging features both at presentation and during follow-up in children with acquired demyelination syndromes.

## Introduction

Antibodies to myelin oligodendrocyte glycoprotein (MOG), detected by live cell-based assays, are found in a proportion of patients with acquired demyelinating syndromes (ADSs) of the central nervous system from early childhood through late adulthood. Since some of these MOG antibody–positive individuals will remain monophasic while others will relapse, identifying those destined for recurrent disease has important implications for both prognosis and treatment decisions. This is particularly relevant in the pediatric age group, where MOG antibodies can be found frequently (up to 40% of those with ADSs),^1^ with studies variably reporting subsequent relapsing disease in 36% to 61% of cases.^2,3,4,5,6,7,8,9^ While it has been suggested that persistent MOG antibody seropositivity following ADS is associated with relapsing disease in both children and adults,^6,10,11^ the studies to date assessing serial serologic MOG antibody status may have preferentially selected patients prone to relapse, resulting in an overestimation of relapse risk. Here, we report on the long-term clinical and imaging outcomes and their association with serial serum MOG antibody measurements in a large unselected cohort of children with ADS followed prospectively from clinical onset.

## Methods

### Participants

**Figure 1.**
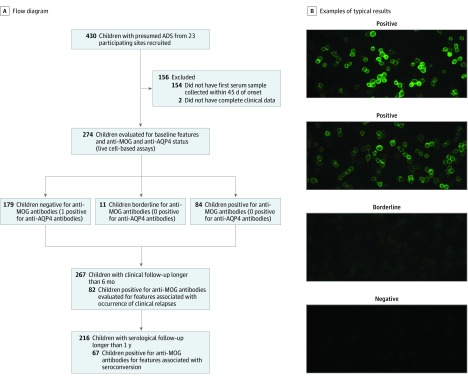
Study Design A, A total of 2022 serial samples from 274 participants with acquired demyelinating syndrome (ADS) with complete clinical data were tested for anti–myelin oligodendrocyte glycoprotein (MOG) IgG1 and anti–aquaporin 4 (AQP4) antibodies. Seven participants were recruited after July 2014, at which time the protocol entered a new phase, requiring participants to meet McDonald 2010 diagnostic criteria. None of these 7 patients were positive for anti-MOG antibodies. B, Example images of typical positive, borderline, and negative results for MOG IgG1 antibody testing.

The Canadian Pediatric Demyelinating Disease Study recruited and longitudinally observed a total of 430 children presenting with a first clinical episode consistent with ADS between July 2004 and February 2017. Of these, all participants for whom a first serum sample was available within 45 days of presentation (n = 274) contributed to the current study (Figure 1A). All participants underwent comprehensive clinical assessments and were offered brain magnetic resonance imaging (MRI) scans and collection of serum samples at the time of clinical presentation; at 3, 6, and 12 months postenrollment; and annually thereafter. Clinical attacks or new MRI lesions were considered evidence of new disease activity if occurring more than 30 days from onset or 90 days in case of an acute disseminated encephalomyelitis (ADEM) presentation.^12^ Data were locked as of May 2018, and participant status was adjudicated based on information available as of their most recent examination to confer a diagnosis of either monophasic ADS (defined as the absence of new clinical attacks and of any MRI evidence of new disease activity on all serial examinations) or of multiple sclerosis (MS), neuromyelitis optica spectrum disorder, or relapsing non-MS disease, using established criteria.^12,13,14^ Guardians and participants provided written informed consent. Younger children provided verbal assent. The study was approved by The Hospital for Sick Children and the research ethics boards of all participating institutions.

### Clinical Characteristics

Clinical features at presentation were recorded, including the presence of signs and symptoms indicating optic neuritis (ON) or transverse myelitis (TM) and those meeting the criteria for ADEM.^12^ Clinical course was evaluated in terms of time from ADS onset to first relapse, annualized relapse rate over the first 4 years (computed including the presenting episode), and relapse phenotype. Disability outcome was recorded using extrapolated Expanded Disability Status Scale (EDSS) scores, as described previously.^15^ The functional system score for visual function was also evaluated. To ensure that EDSS and visual functional system scores could be compared at a consistent time from onset across all participants, we selected the scores obtained at 4 years following clinical presentation (the median postonset observation time of the cohort). Dates and durations of treatment with disease-modifying therapies were recorded.

### MOG Antibody Status and Cerebrospinal Fluid Studies

Myelin oligodendrocyte glycoprotein antibody status was assessed in 2022 serum samples. Of these, 5 samples obtained within 30 days of plasma exchange were excluded. All samples were tested using a live MOG IgG1-specific cell-based assay^16^ and scored semiquantitatively by a single blinded operator (Figure 1B). Any positive sample was titrated using antihuman IgG (H^+^L) as detecting antibody. Results of the IgG1 assay in samples obtained within 45 days from clinical onset were used to determine the serologic status at presentation. For assessment of serial samples, subsequent samples with a titer of 1:200 or greater were considered seropositive. All samples were also screened using a live cell-based aquaporin 4 (AQP4) assay as a specificity control.^17^ Where indicated, presence of cerebrospinal fluid oligoclonal bands was assessed by isoelectric focusing as part of clinical evaluation.

### MRI Analysis

Research MRI scans included axial and sagittal T2-weighted images, fluid-attenuated inversion recovery images, T1-weighted images, and T1-weighted images after administration of gadolinium. Scans acquired within 45 days of clinical presentation were analyzed by applying a standardized scoring tool^18^ modified to include additional MRI variables^19^ and blinded to MOG status and to clinical outcome.

Serial scans were analyzed for the occurrence of new T2 lesions and lesion resolution. T2 lesions were segmented using a bayesian classifier and then manually corrected by a trained expert. T1 lesion volumes were automatically segmented as a subset of T2 lesion voxels with T1 intensity less than 87% of the normal-appearing white matter. When available, clinical scans obtained apart from the research protocol were also evaluated for the presence of new lesions.

### Statistical Analysis

Figure 1A summarizes the study design. First, presenting clinical, MRI, and laboratory features were compared between anti-MOG antibody–positive and anti-MOG antibody–negative participants, as defined by the result of their baseline sample. The analysis was also repeated separately in participants older and younger than 11 years at onset and in participants with or without ADEM at presentation. We then compared the presenting clinical and MRI features of participants who were persistently positive for anti-MOG antibodies (defined as anti-MOG antibody–positive results in all serial samples up to and including at least 1 year) with those who became negative for anti-MOG antibodies. To minimize confounding of missing values and different lengths of follow-up, we conducted an additional sensitivity analysis focusing on participants for whom samples were available at all time points of baseline, 3 months, 1 year, and 2 years.

Clinical and MRI courses were compared between participants positive and negative for anti-MOG antibodies with a minimum follow-up of 6 months (except for annualized relapse rate and EDSS, which were further restricted to participants observed for a minimum of 4 years). The analysis was repeated after excluding participants with a diagnosis of MS. The presenting features were then compared between anti-MOG antibody–positive participants with monophasic or relapsing course and used as inputs for a random forest classifier (1000 trees; Python SKLearn). A backward elimination process^20^ was applied to identify features that could work in combination to predict a relapsing outcome, with the feature with the lowest Gini impurity removed from the input at each repetition.

Feature comparisons between groups were performed using χ^2^ or Fisher exact tests for categorical variables and Mann-Whitney *U* tests for continuous variables. The correlation between continuous variables was assessed by Spearman rank correlation. A *P* value less than .05 was considered statistically significant, and all *P *values were 2-tailed. Time to conversion to seronegative status was plotted using the Kaplan-Meier method. Analyses were performed using Python version 3.6.5 (Python Software Foundation) and R version 3.1.3 (The R Foundation).

## Results

### Presenting Features of the ADS Cohort

The presence of MOG and AQP4 antibodies was assessed in 274 children with ADS, of whom 140 (51.1%) were female, and the median (interquartile range [IQR]) age of all participants was 10.8 (6.2-13.9) years (Table 1). Myelin oligodendrocyte glycoprotein antibodies were detected in 84 participants (30.7%), while 179 (65.3%) tested negative and 11 (4.0%) had borderline results. Aquaporin 4 antibodies were detected in 1 participant, who was negative for anti-MOG antibodies. Anti-MOG antibody–positive participants were younger than anti-MOG antibody–negative participants, and 81 of 84 (96%) presented with ON, TM, ADEM, or a combination of these.

**Table 1.** Demographic, Clinical, Imaging, and Cerebrospinal Fluid (CSF) Laboratory Features at Presentation by Initial Myelin Oligodendrocyte Glycoprotein Status in All Participants and in Participants Stratified by Age at Clinical Onset

Characteristic	No./Total No. (%)										
	All Participants					<11 y			≥11 y		
	Total	Borderline	Seropositive	Seronegative	*P* Value^a^	Seropositive	Seronegative	*P* Value^a^	Seropositive	Seronegative	*P* Value^a^
**Demographic and Clinical Features at Presentation**											
Participants, No.	274	11	84	179	NA	65	69	NA	19	110	NA
Time from onset to first serum procurement, median (IQR), d	9 (5-19)	22 (12-28)	10 (5-20)	9 (5-17)	.23	10 (6-21)	8 (4-15)	.06	12 (4-18)	10 (5-18)	.33
Female	140/274 (51.1)	7/11 (64)	46/84 (55)	87/179 (48.6)	.42	38/65 (58)	30/69 (43)	.12	8/19 (42)	57/110 (51.8)	.59
Age at clinical onset, median (IQR), y	10.80 (6.18-13.87)	6.18 (5.44-11.32)	7.31 (4.93-10.57)	12.41 (8.09-14.43)	<.001	6.27 (4.44-8.65)	6.31 (2.79-9.57)	.46	13.31 (12.62-14.35)	14.04 (12.82-15.31)	.06
Duration of clinical follow up, median (IQR), y	6.68 (4.24-8.86)	6.92 (4.36-8.05)	6.74 (4.77-8.75)	6.35 (4.08-8.99)	.36	6.91 (4.79-8.45)	7.56 (4.23-9.01)	.32	6.68 (4.18-9.08)	6.03 (4.07-8.69)	.42
Presenting phenotype^b^											
ADEM	67/274 (24.5)	5/11 (45)	32/84 (38)	30/179 (16.8)	<.001	29/65 (45)	22/69 (32)	.18	3/19 (16)	8/110 (7.3)	.21
ADEM with ON	3/274 (1.1)	0	3/84 (4)	0	.03	2/65 (3)	0	.23	1/19 (5)	0	.15
ADEM with TM	10/274 (3.6)	0	9/84 (11)	1/179 (0.6)	<.001	9/65 (14)	0	.001	0	1/110 (0.9)	>.99
ADEM with ON and TM	2/274 (0.7)	1/11 (9)	1/84 (1)	0	.32	1/65 (2)	0	.49	0	0	>.99
ON											
Monofocal	68/274 (24.8)	3/11 (27)	32/84 (38)	33/179 (18.4)	<.001	21/65 (32)	10/69 (14)	.03	11/19 (58)	23/110 (20.9)	.002
Polyfocal	12/274 (4.4)	1/11 (9)	2/84 (2)	9/179 (5.0)	.51	0	2/69 (3)	.50	2/19 (11)	7/110 (6.4)	.62
TM											
Monofocal	53/274 (19.3)	0	7/84 (8)	46/179 (25.7)	.002	6/65 (9)	14/69 (20)	.12	1/19 (5)	32/110 (29.1)	.04
Polyfocal	15/274 (5.5)	0	5/84 (6)	10/179 (5.6)	.87	4/65 (6)	6/69 (9)	.75	1/19 (5)	4/110 (3.6)	.56
ON with TM	4/274 (1.5)	0	3/84 (4)	1/179 (0.6)	.04	2/65 (3)	0	.49	1/19 (5)	1/110 (0.9)	.27
Other	55/274 (20.1)	2/11 (18)	3/84 (4)	50/179 (27.9)	<.001	3/65 (5)	15/69 (22)	.005	0	35/110 (31.8)	.002
**MRI and CSF Laboratory Features at Presentation**^**c**^											
Brain lesions											
Patients with lesions	172/253 (68.0)	9/11 (82)	51/76 (67)	112/166 (67.5)	>.99	40/57 (70)	44/67 (66)	.70	11/19 (58)	68/99 (69)	.52
Count, median (IQR)^d^	3 (0->15)	8 (0.75->15)	5.5 (0->15)	2 (0->15)	.13	14 (0->15)	2 (0-13)	.02	1 (0-4.5)	4 (0->15)	.07
Excluding patients without lesions	12 (3->15)	>15 (6->15)	>15 (5.5->15)	8 (2->15)	.02	>15 (10.75->15)	7 (2->15)	.001	4 (2-12)	9 (3->15)	.07
Presence of ≥1 discrete lesions	117/172 (68.0)	2/9 (22)	27/51 (53)	88/112 (78.6)	.002	18/40 (45)	23/44 (52)	.65	9/11 (82)	65/68 (96)	.14
Only well-defined lesions	83/172 (48.3)	1/9 (11)	14/51 (27)	68/112 (60.7)	<.001	7/40 (18)	16/44 (36)	.09	7/11 (64)	52/68 (76)	.46
Diffuse bilateral pattern	61/172 (35.5)	6/9 (67)	31/51 (61)	24/112 (21.4)	<.001	28/40 (70)	18/44 (41)	.01	3/11 (27)	6/68 (9)	.11
≥1 Cerebellar lesions	67/172 (39.0)	5/9 (56)	22/51 (43)	40/112 (35.7)	.46	19/40 (48)	17/44 (39)	.55	3/11 (27)	23/68 (34)	>.99
≥1 Cerebellar peduncle lesions	52/172 (30.2)	2/9 (22)	20/51 (39)	30/112 (26.8)	.16	14/40 (35)	13/44 (30)	.76	6/11 (55)	17/68 (25)	.10
≥1 Brainstem lesions	103/172 (59.9)	7/9 (78)	34/51 (67)	62/112 (55.4)	.23	25/40 (62)	27/44 (61)	.91	9/11 (82)	35/68 (51)	.10
≥1 Periventricular lesions	98/172 (57.0)	4/9 (44)	30/51 (59)	64/112 (57.1)	.98	25/40 (62)	17/44 (39)	.049	5/11 (45)	47/68 (69)	.23
≥3 Periventricular lesions	59/172 (34.3)	3/9 (33)	19/51 (37)	37/112 (33.0)	.73	18/40 (45)	6/44 (14)	.003	1/11 (9)	31/68 (46)	.02
≥1 Lesion perpendicular to major axis of corpus callosum	53/172 (30.8)	2/9 (22)	7/51 (14)	44/112 (39.3)	.002	3/40 (8)	5/44 (11)	.72	4/11 (36)	39/68 (57)	.21
≥1 Basal ganglia lesions	37/172 (21.5)	4/9 (44)	17/51 (33)	16/112 (14.3)	.009	17/40 (42)	9/44 (20)	.05	0	7/68 (10)	.58
≥1 Thalamic lesions	57/172 (33.1)	4/9 (44)	30/51 (59)	23/112 (20.5)	<.001	27/40 (68)	13/44 (30)	.001	3/11 (27)	10/68 (15)	.38
≥1 Juxtacortical lesions	116/172 (67.4)	8/9 (89)	42/51 (82)	66/112 (58.9)	.006	37/40 (92)	24/44 (55)	<.001	5/11 (45)	42/68 (62)	.49
≥1 T1 hypointense lesions	77/172 (44.8)	1/9 (11)	15/51 (29)	61/112 (54.5)	.005	15/40 (38)	12/44 (27)	.44	0	49/68 (72)	<.001
≥1 Lesion enhancement	47/149 (31.5)	2/7 (29)	5/42 (12)	40/100 (40.0)	.002	5/34 (15)	7/38 (18)	.92	0	33/62 (53)	.006
≥1 Gadolinium-negative T1 hypointense lesions	54/149 (36.2)	0	11/42 (26)	43/100 (43.0)	.09	11/34 (32)	7/38 (18)	.28	0	36/62 (58)	.002
OCBs	47/169 (27.8)	0	8/49 (16)	39/113 (34.5)	.02	6/38 (16)	8/41 (20)	.89	2/11 (18)	31/72 (43)	.19

Abbreviations: ADEM, acute disseminated encephalomyelitis; IQR, interquartile range; MRI, magnetic resonance imaging; NA, not applicable; OCB, oligoclonal band; ON, optic neuritis; TM, transverse myelitis.

^a^ *P* values are computed based on comparisons between seropositive and seronegative participants. Participants with borderline results were not considered in the comparisons of participants stratified by age.

^b^ For each participant, the clinical presentation was classified as monofocal if all clinical deficits were localizable to a single central nervous system site, while findings implicating more than 1 central nervous system location were classified as polyfocal. Magnetic resonance imaging scans were not used to define monofocal or polyfocal designations.

^c^ Brain MRI scans acquired within 45 days from onset were available in 253 participants. The frequencies of all features pertaining to lesion aspect and location were computed only among patients with brain lesions at baseline. The denominator for each feature corresponds to the total number of participants in which that feature was evaluated. Analyses of lesion enhancement and gadolinium-negative T1 hypointense lesions were further restricted to participants who had gadolinium administered.

^d^ The total number of T2 lesions were estimated by manual count, with lesion numbers exceeding 15 listed as greater than 15.

Several clinical and MRI features differed by age at presentation. Consistent with the common presentation of ADEM, anti-MOG antibody–positive participants younger than 11 years had a high number of lesions that were often ill defined and/or distributed in a diffuse bilateral pattern, with frequent thalamic and juxtacortical involvement. Conversely, anti-MOG antibody–positive participants older than 11 years presented with fewer focal lesions, and 8 of 19 (42%) (all presenting with ON) had normal brain MRI findings at onset. When present in older anti-MOG antibody–positive participants, brain lesions more frequently showed well-defined borders. The full description of clinical, MRI, and laboratory features relative to MOG status at presentation is presented in Table 1, and a comparison between participants with and without ADEM is reported in eTable 1 in the Supplement.

### Temporal Evolution of Serologic Status

**Figure 2.**
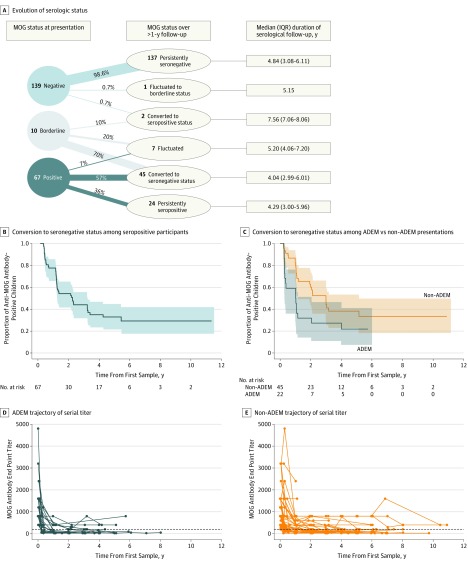
Myelin Oligodendrocyte Glycoprotein (MOG) Results on Serial Samples A, Evolution of serologic status in participants with follow-up greater than 1 year. Only 2 of 139 participants who were negative for anti-MOG antibodies at presentation changed serological status in subsequent examinations: 1 participant became seropositive at 3 months and was persistently positive for the subsequent 8 years and 1 had an isolated finding of a borderline result at 2 years from onset. Of the 10 participants with borderline results at onset, 7 became seronegative, 2 fluctuated between negative and borderline status and 1 became seropositive in the last follow-up sample (7 years from onset; titer, 1:200). B, Kaplan-Meier curve for the time to conversion to seronegative status in all participants who were positive for anti-MOG antibodies at time of presentation. The shaded areas indicate 95% CIs. C, Kaplan-Meier curves in participants with acute disseminated encephalomyelitis (ADEM) vs non-ADEM presentations. Participants with ADEM had a considerably shorter time to seroconversion than participants without ADEM. The shaded areas indicate 95% CIs. D and E, Trajectory of serial MOG titers of anti-MOG antibody–positive participants with ADEM at onset (D) and without ADEM at onset (E). The data points indicate the titers measured at each serological evaluation.

The evolution of serological status was evaluated in 216 participants with samples available for follow-up more than 1 year after baseline (median [IQR] follow-up, 4.70 [3.07-6.11] years) (Figure 2A). A total of 137 of 139 participants (98.6%) who were negative for anti-MOG antibodies at presentation remained negative in all subsequent examinations.

Of the 67 participants who were positive for anti-MOG antibodies at onset and observed for at least 1 year from presentation, 24 (36%) remained persistently positive, 5 (7%) fluctuated between positive and negative status, and 38 (57%) became seronegative (median [IQR] time to conversion, 1.02 [0.32-1.75] years) (Figure 2B). Of 22 initially anti-MOG antibody–positive participants with ADEM with at least 1 year of follow-up, 17 (77%) became seronegative after a median (IQR) of 4.63 (3.45-12.71) months (Figure 2C). Participants who were persistently seropositive tended to be older at presentation compared with those who converted to seronegative status (median [IQR] age, 9.06 [6.60-13.36] years vs 6.95 [5.28-9.96] years; *P* = .03) and were more likely to have presented with monofocal ON (14 of 24 [58%] vs 9 of 38 [24%]; *P* = .01) (eTable 2 in the Supplement).

Myelin oligodendrocyte glycoprotein titers showed a global decrease over follow-up that was steeper in the first year (Figure 2D). These changes in MOG antibody titers were unlikely to be influenced by treatment, as only 2 of 84 participants (2%) who were positive for anti-MOG antibodies at the time of initial presentation were ever exposed to disease-modifying therapies during their follow-up. In the sensitivity analysis of the 126 participants for whom all samples at presentation, 3 months, 1 year, and 2 years were available, the proportion of participants who converted to seronegative status was consistent with findings observed in the larger cohort (eFigure 1 in the Supplement).

### Clinical Disease Course

#### Diagnostic Outcome as a Function of MOG Status at Presentation

During the prospective follow-up, 66 participants met McDonald 2010 diagnostic criteria for MS, while 11 were diagnosed as having a relapsing non-MS disease, 1 as having AQP4-positive neuromyelitis optica spectrum disorder, and 196 as having a monophasic course. Among the 66 children who eventually met diagnostic criteria for MS, 11 (17%) were positive for anti-MOG antibodies at presentation. Despite meeting formal MS diagnostic criteria, all 11 of these participants had clinical and MRI features that were considered by the study clinicians to be atypical for MS (eg, clinical attacks largely restricted to ON, near-complete resolution of initial T2 lesions, no or few small lesions on follow-up scans). At the time of the most recent evaluation of these 11 patients (mean [range] follow-up from initial presentation, 8.96 [1.69-12.13] years), none of these participants were considered by their treating clinician to have a clinical disease course consistent with MS.

Myelin oligodendrocyte glycoprotein antibodies were found in almost all participants eventually diagnosed as having relapsing non-MS disease (10 of 11 [91%]) and in 63 of 196 participants with monophasic ADS (32.1%). Of the 10 anti-MOG antibody–positive children with relapsing non-MS disease, 6 had relapsing ON, 1 had relapsing TM, and 3 had multiple neurological relapses (including either ON or TM and other neurological deficits) following initial ADEM presentation.

#### Clinical and MRI Disease Activity Outcomes as a Function of MOG Status at Time of Initial Presentation

Clinical relapses occurred in similar proportions of patients positive and negative for anti-MOG antibodies (53 of the latter met criteria for MS) observed for a minimum of 6 months from presentation (Table 2). When excluding participants who met diagnostic criteria for MS, the occurrence of clinical relapses was more common among anti-MOG antibody–positive participants (9 of 71 [13%] vs 2 of 121 [2%]; *P* = .002), ie, 10 of 11 participants with relapsing non-MS disease (91%) were positive for anti-MOG antibody at presentation. Relapses in anti-MOG antibody–positive participants were more commonly restricted to the optic nerve or spinal cord. A total of 5 of 20 anti-MOG antibody–positive participants (25%) who presented with monofocal ON and had normal findings on brain MRI had subsequent clinical relapses compared with none of the 17 anti-MOG antibody–negative children with the same presentation (*P* = .049). Additionally, 32 children who presented with monofocal TM had normal findings on brain MRI at onset (3 anti-MOG antibody–positive and 29 anti-MOG antibody–negative children), only 1 of whom (who was negative for anti-MOG antibodies) experienced a relapse (TM). Among participants presenting with ADEM, clinical relapses occurred in 3 of 32 participants (9%) who were positive for anti-MOG antibodies at presentation vs 1 of 30 (3%) who were seronegative. While EDSS scores were comparably low across seropositive and seronegative participants at 4 years of follow-up (Table 2), anti-MOG antibody–positive participants with a monophasic disease course had lower EDSS scores at 4 years than monophasic anti-MOG antibody–negative participants (median [IQR] score, 0 [0-1] vs 0 [0-1.5]; *P* = .007). This difference did not persist when the comparison was restricted to participants with the same clinical presentation of monofocal ON, monofocal TM, or ADEM.

**Table 2.** Clinical and Magnetic Resonance Imaging (MRI) Disease Activity as a Function of Baseline Myelin Oligodendrocyte Glycoprotein Status in Participants With More Than 6 Months’ Follow-up

Characteristic	No./Total No. (%)							
	Total				With Non-MS Disease			
	Borderline	Seropositive	Seronegative	*P* Value^a^	Borderline	Seropositive	Seronegative	*P* Value^a^
Participants, No.^b^	11	82	174	NA	10	71	121	NA
Duration of clinical follow-up, median (IQR), y	6.92 (4.63-8.05)	6.86 (4.79-8.87)	6.78 (4.30-9.01)	.38	6.41 (4.37-8.08)	6.68 (4.76-8.23)	7.00 (4.66-8.98)	.28
Clinical diagnosis								
MS	1/11 (9)	11/82 (13)^c^	53/174 (30.5)	.005	NA	NA	NA	NA
Relapsing non-MS disease	0	10/82 (12)^c^	1/174 (0.6)	<.001	0	10/71 (14)	1/121 (0.8)	<.001
NMOSD	0	0	1/174 (0.6)	>.99	0	0	1/121 (0.8)	>.99
Monophasic ADS	10/11 (91)	61/82 (74)	119/174 (68.4)	.40	10/10 (100)	61/71 (86)	119/121 (98.3)	.001
Exposure to DMT	1/11 (9)	2/82 (2)	42/174 (24.1)	<.001	0	0	0	>.99
Clinical relapses	0	16/82 (20)	39/174 (22.4)	.72	0	9/71 (13)	2/121 (1.7)	.002
Time to second attack, median (IQR), y	NA	0.92 (0.31-1.60)	0.83 (0.41-1.69)	.47	NA	1.43 (0.52-2.12)	0.81 (0.36-1.25)	.28
Relapse								
ON only	NA	10/16 (63)	1/39 (3)	<.001	NA	7/9 (78)	0	.11
TM only	NA	1/16 (6)	2/39 (5)	>.99	NA	0	1/2 (50)	.18
ON or TM with other	NA	5/16 (31)	14/39 (36)	.40	NA	2/9 (22)	1/2 (50)	.49
Other	NA	0	22/39 (56)	<.001	NA	0	0	>.99
ARR in the first 4 y, median (IQR)^d^	NA	0.50 (0.50-0.75)	0.75 (0.75-1.00)	.18	NA	0.50 (0.50-0.75)	0.75 (0.63-0.88)	.40
EDSS score at 4 y, median (IQR)^e^	0 (0-1.50)	0 (0-1.00)	1.00 (0-1.50)	.049	0 (0-0.38)	0 (0-1.00)	0 (0-1.50)	.02
Visual functional system score at 4 y, median (IQR)^f^	0 (0-1.00)	0 (0-1.00)	0 (0-0)	.04	0 (0-0.25)	0 (0-0)	0 (0-0)	.18
MRI follow-up								
Duration of MRI follow-up, median (IQR), y	5.03 (1.50-5.54)	4.04 (1.11-5.98)	3.43 (1.04-5.98)	.34	4.54 (1.32-5.13)	3.98 (1.10-5.93)	3.01 (1.03-5.97)	.37
No. of scans per participant, median (IQR)	5 (3-6)	5 (3-8)	5 (3-8)	.28	5 (3-5)	5 (3-8)	5 (3-7)	.38
Complete brain lesion resolution	5/8 (62)	26/49 (53)	20/109 (18.3)	<.001	5/7 (71)	25/42 (60)	20/62 (32)	.009
New MRI lesions^g^	1/10 (10)	15/78 (19)	56/162 (34.6)	.03	0	5/67 (7)	8/112 (7.1)	.85
Lesion volume at last scan, median (IQR), cm^3^								
Total T2	0.19 (0-0.38)	0 (0-0.03)	0.22 (0-3.41)	.006	0 (0-0.19)	0 (0-0.01)	0 (0-0.12)	.18
Total T1	0 (0-0.01)	0 (0-0)	0 (0-1.01)	.01	0 (0-0)	0 (0-0)	0 (0-0)	.17

Abbreviations: ADS, acquired demyelinating syndrome; ARR, annualized relapse rate; DMT, disease-modifying treatment; EDSS, Expanded Disability Status Scale; IQR, interquartile range; MS, multiple sclerosis; NA, not applicable; NMOSD, neuromyelitis optica spectrum disorder; ON, optic neuritis; TM, transverse myelitis.

^a^ *P* values are computed based on comparisons between seropositive and seronegative participants.

^b^ Six participants with a diagnosis of monophasic ADS (2 seropositive and 4 seronegative patients) and 1 seronegative participant diagnosed as having MS had less than 6 months of clinical follow-up and are not included here.

^c^ Of the 11 seropositive patients with a diagnosis of MS, 7 experienced clinical relapses and 4 were diagnosed as having MS based on evidence of new lesions on MRI. Of the 10 seropositive participants with a diagnosis of relapsing non-MS disease, 9 experienced clinical relapses not meeting the current MS diagnostic criteria and 1 developed new, asymptomatic lesions 3 years after an ADEM presentation.

^d^ ARR is computed among participants with relapsing disease, including the presenting episode.

^e^ EDSS score at 4 years from presentation was evaluated in 65 seropositive and 123 seronegative participants.

^f^ Visual functional system score at 4 years from presentation was evaluated in 62 seropositive and 116 seronegative participants.

^g^ Serial brain MRI scans for more than 6 months from presentation were available in 78 seropositive and 162 seronegative participants.

Serial brain MRI scans revealed that participants who were positive for anti-MOG antibodies at presentation were more likely to exhibit either complete resolution of all baseline lesions or smaller T2 and T1 total lesion volumes on their last MRI (Table 2). Among the presenting features of anti-MOG antibody–positive participants, older age was identified by the random forest analysis as the feature that was most strongly associated with a relapsing outcome, with a Gini impurity-derived importance 3-fold and 4-fold greater than the following 2 features in the ranking (T1 hypointense lesions and lesion count, respectively) (eTable 3 in the Supplement).

#### Clinical Relapses as a Function of Serial MOG Status

**Figure 3.**
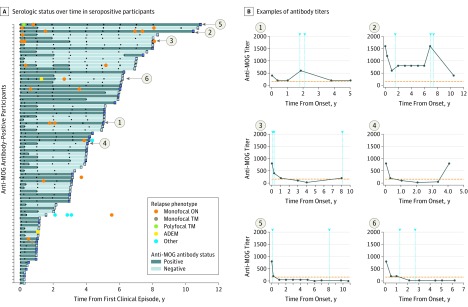
Evolution of Serologic Status and Clinical Relapses in Participants Who Were Positive for Anti–Myelin Oligodendrocyte Glycoprotein (MOG) Antibodies at Time of Initial Presentation A, Serologic status over time in 77 anti-MOG antibody–positive participants with serial samples. Each bar represents an individual participant, and the square at the end of each bar indicates the serological status in the last follow-up sample. Dark blue bars indicate seropositive status, and light blue bars indicate seronegative status. Black dots represent the time of sampling. Colored circles indicate clinical relapses. B, Representative examples of antibody titers in participants during follow-up. Graphs 1 and 2 show 2 cases in which transient antibody titer increases were associated with clinical relapses (arrowheads). Graphs 3 and 4 show patients with reappearance of MOG antibodies after initial seroconversion from seropositive to seronegative status, in one case associated with a clinical attack. Graphs 5 and 6 show examples of patients who experienced clinical relapses after conversion to seronegative status.

Of the 16 participants who were positive for anti-MOG antibodies at presentation who experienced clinical relapses, 9 (56%) were persistently seropositive (accounting for 28% of all persistently seropositive participants), 2 (13%) had fluctuating serologic status, and 5 (31%) converted to seronegative status (Figure 3A) (eFigure 2 in the Supplement). Of the latter, 4 participants experienced at least 1 relapse after detection of the first seronegative sample without being exposed to disease-modifying therapies prior to or at the time of the relapse.

A monophasic course was observed in most participants with an ADEM presentation, including 3 of 5 (60%) who were persistently seropositive, 27 of 28 (96%) who were seronegative, and 17 of 18 (94%) who converted to seronegative status. The 1 patient who relapsed after seroconversion had multiple relapses.

While our protocol did not include sampling at the time of relapse, all of the samples acquired within 3 months of a clinical relapse in participants who were positive for anti-MOG antibodies at presentation were also seropositive at collection. One participant who was seropositive at presentation who then became seronegative experienced a subsequent relapse at the time of a planned study visit; findings from serum analysis at that time demonstrated re-emergence of MOG antibodies (Figure 3).

## Discussion

In this prospective cohort of pediatric patients with incident ADS, we serially evaluated MOG serostatus from clinical onset, with rigorous longitudinal clinical and MRI assessments extending for up to 13 years. Several of our findings reinforce and extend prior reports, showing that MOG antibodies are common at onset in pediatric ADS,^2,21^ particularly among younger children and those who present with ADEM or ON.^3,22,23,24^ We also confirmed a bimodal distribution of clinical presentations and MRI features in anti-MOG antibody–positive children, with younger patients being more likely to present with ADEM and/or a large number of ill-defined MRI lesions and older children more often presenting with ON and no or few well-defined brain lesions.^2,25^

A strength of our study was the procurement of serial samples at predefined intervals, enabling us to demonstrate that a negative result for MOG antibodies in proximity to the ADS presentation almost entirely excludes subsequent positive results over time. The absence of MOG antibodies at onset has key clinical implications. First, in anti-MOG antibody–negative children with isolated ON or TM (ie, with normal findings on brain MRI and no clinical features suggesting lesions in the brain) and in anti-MOG antibody–negative patients with ADEM, a monophasic outcome is highly likely. Second, anti-MOG antibody–negative patients with clinical, MRI, and cerebrospinal fluid findings meeting McDonald MS diagnostic criteria follow a typical MS disease course. The circumstance of MOG antibodies identified in children meeting MS diagnostic criteria requires specific comment. Careful review of all such patients in our cohort demonstrated a clinical and imaging disease evolution atypical of MS. As such, we agree with previous authors^16,26^ advocating that the presence of MOG antibodies “plead against MS diagnosis.”^21^

Most patients who were seropositive at time of initial presentation subsequently became seronegative (after a median of 1 year from onset) and experienced a monophasic clinical course. Although most relapses in patients who were initially seropositive occurred while they were seropositive, nearly 40% of these patients experienced 1 or more clinical relapses after their first seronegative result. Of these, 4 of 16 participants (25%) were persistently seronegative, while 2 of 16 (13%) had anti-MOG antibodies again detected in subsequent samples. It is notable that we did not collect samples at the time of relapse and, as such, we may have missed more dynamic changes in serostatus; however, when serum was obtained within 3 months before or following a clinical attack in participants with prior seropositive status, MOG antibodies were reliably detected. Defining the optimal frequency of sampling required to more formally assess the relationship between an acute relapse and continued presence or re-emergence of MOG antibodies will require frequent regular sampling as well as sampling at time of relapse. It should also be noted that 72% of persistently seropositive patients in our cohort have not experienced a second attack despite prolonged observation. Among all presenting features of the anti-MOG antibody–positive patients evaluated in our study, an older age at presentation was most strongly associated with a relapsing outcome.

In line with previous reports,^27,28,29^ most anti-MOG antibody–positive children in our cohort had a favorable outcome, with monophasic courses for more than 80% of patients, low EDSS scores at last follow-up, and either complete resolution or very low residual lesion volumes at last brain MRI scan. However, the extended long-term outcome for such patients remains to be determined.

Based on our findings, we would advocate that MOG testing has relevance for all children with ADS. Negative testing at onset provides reassurance for families of a likely monophasic illness if their child has presented with isolated ON, TM, or ADEM. Furthermore, a positive MOG result in a pediatric patient is associated with a non-MS disease course.

Serial serological surveillance is valuable in patients found to be seropositive at presentation, whereas it is likely to be of little clinical utility in patients identified as seronegative at presentation. Conversion to seronegative status was associated with lesser risk of subsequent relapse, although it does not entirely preclude it. In turn, persistent seropositivity is not necessarily associated with disease relapse. If serial testing following presentation is limited by resources, then a 12-month time point following initial presentation will identify more than half of the patients destined to become seronegative, including most patients with ADEM likely to convert.

Given that many of the children found to be positive for anti-MOG antibodies at presentation will remain monophasic, we would not advocate broad institution of long-term immunomodulatory therapy following their initial presentation. In contrast, anti-MOG antibody–positive patients who do relapse are typically offered treatment with immunoglobulins, anti-CD20, or more general immunosuppressive therapies.^7,30^ Further research is required to confirm whether relapsing MOG demyelination is a lifelong illness and to define the morbidity of seropositive-related relapses more fully. In particular, future studies should aim to quantify the effect of seropositive-related demyelination on age-expected brain growth and white matter maturation, both of which are negatively affected in MS,^19,31,32,33^ and on cognitive performance. All of this information is essential to better inform the risk-to-benefit ratio of long-term immunosuppression.

### Limitations

This study had limitations. The study was not designed to acquire serological samples at time of relapses and might have therefore not captured transient changes in serological status associated with reoccurrence of either clinical or subclinical disease activity. Future studies, with frequent acquisition of serological samples following a first clinical episode and at the time of subsequent attacks, are needed to fully clarify the dynamic changes in serostatus in patients initially seropositive for anti-MOG antibodies. Additionally, since this study was originally designed for the study of patients with possible ADSs, patients with encephalitis-like presentations, also described in association with anti-MOG antibodies, would not have been included in our cohort.

## Conclusions

Anti MOG-antibodies are common in children with ADSs and are transient in approximatively half of cases. Most anti-MOG antibody–positive children experience a monophasic disease, with clinical relapses occurring more commonly, although not exclusively, in children with persistent seropositivity. Overall, most anti-MOG antibody–positive children have a favorable outcome, and the presence of anti-MOG antibodies at the time of incident demyelination should not immediately prompt the initiation of long-term immunomodulatory therapy.

## Notes

Supplement. **eTable 1.** Baseline clinical, MRI, and laboratory features in patients with and without ADEM. **eTable 2.** Baseline clinical and MRI features of persistently seropositive patients, patients converting to seronegative status, or patients with fluctuating serological status. **eTable 3.** Ranking of baseline clinical, MRI, and laboratory features most strongly associated with relapses in seropositive children. **eFigure 1.** Sensitivity analysis for evolution of serological status. **eFigure 2.** Kaplan-Meier curve for risk of a second clinical attack in participants persistently seropositive vs those who converted to seronegative status.
