# Supplementary material for: Serial Anti–Myelin Oligodendrocyte Glycoprotein Antibody Analyses and Outcomes in Children With Demyelinating Syndromes
Source: JAMA Neurol. 2019 Sep 23;77(1). doi: 10.1001/jamaneurol.2019.2940 (PMC6763982; doi:10.1001/jamaneurol.2019.2940)
Supplement: Supplement. — eTable 1. Baseline clinical, MRI, and laboratory features in patients with and without ADEM. eTable 2. Baseline clinical and MRI features of persistently seropositive patients, patients converting to seronegative status, or patients with fluctuating serological status. eTable 3. Ranking of baseline clinical, MRI, and laboratory features most strongly associated with relapses in seropositive children. eFigure 1. Sensitivity analysis for evolution of serological status. eFigure 2. Kaplan-Meier curve for risk of a second clinical attack in participants persistently seropositive vs those who converted to seronegative status. [file jamaneurol-77-82-s001.pdf]

Supplementary Online Content

Waters P, Fadda G, Woodhall M, et al; Canadian Pediatric Demyelinating Disease Network. Serial anti–myelin oligodendrocyte glycoprotein antibody analyses and outcomes in children with demyelinating syndromes. *JAMA Neurol*. Published online September 23, 2019. doi:10.1001/jamaneurol.2019.2940

eTable 1. Baseline clinical, MRI, and laboratory features in patients with and without ADEM
eTable 2. Baseline clinical and MRI features of persistently seropositive patients, patients converting to seronegative status, or patients with fluctuating serological status
eTable 3. Ranking of baseline clinical, MRI, and laboratory features most strongly associated with relapses in seropositive children
eFigure 1. Sensitivity analysis for evolution of serological status
eFigure 2. Kaplan-Meier curve for risk of a second clinical attack in participants persistently seropositive vs those who converted to seronegative status

This supplementary material has been provided by the authors to give readers additional information about their work.

© 2019 Waters P et al. *JAMA Neurology.*

eTable 1. Baseline clinical, MRI, and laboratory features in patients with and without ADEM

	ADEM					NON-ADEM				
	All	Borderline	Positive	Negative	p value	All	Borderline	Positive	Negative	p value
**Participants**	67	5	32	30	1	207	6	52	149	1
Sex (Female)	34 (51)	3 (60)	18 (56)	13 (43)	0.45	106 (51)	4 (67)	28 (54)	74 (50)	0.72
Age at onset	5.84 (3.37-9.75)	5.17 (3.66-5.84)	5.27 (3.76-7.19)	7.03 (3.02-11.45)	0.16	12.20 (8.60-14.12)	9.72 (6.91-11.81)	9.12 (6.50-12.04)	12.88 (9.70-14.61)	<0.0001
Presenting phenotype										
ON	3/67 (4)	0/5 (0)	3/32 (9)	0/30 (0)	0.24	80/207 (39)	4/6 (67)	34/52 (65)	42/149 (28)	<0.0001
TM	10/67 (15)	0/5 (0)	9/32 (28)	1/30 (3)	0.013	68/207 (33)	0/6 (0)	12/52 (23)	56/149 (38)	0.083
ON + TM	2/67 (3)	1/5 (20)	1/32 (3)	0/30 (0)	1	4/207 (2)	0/6 (0)	3/52 (6)	1/149 (1)	0.054
Other	52/67 (78)	4/5 (80)	19/32 (59)	29/30 (97)	0.00051	55/207 (27)	2/6 (33)	3/52 (6)	50/149 (34)	<0.0001
Clinical relapses	4/67 (6)	0/5 (0)	3/32 (9)	1/30 (3)	0.61	51/207 (25)	0/6 (0)	13/52 (25)	38/149 (26)	1
Time to second attack (years)	1.77 (1.34-2.56)	-	2.12 (1.77-3.00)	1.10 (1.10-1.10)	0.19	0.74 (0.36-1.49)	-	0.64 (0.24-1.30)	0.81 (0.40-1.82)	0.19
MRI and laboratory features[a]										
Lesions present	61/62 (98)	5/5 (100)	29/29 (100)	27/28 (96)	0.49	111/191 (58)	4/6 (67)	22/47 (47)	85/138 (62)	0.088
Lesions count	>15 (9.25->15)	13 (3.25->15)	>15(>15->15)	>15 (2->15)	0.0037	1.00 (0.00-9.50)	3.50 (0.25-13.50)	0.00 (0.00-4.00)	2.00 (0.00-11.00)	0.024
≥ 1 Cerebellar lesion	29/62 (47)	3/5 (60)	15/29 (52)	11/28 (39)	0.5	38/191 (20)	2/6 (33)	7/47 (15)	29/138 (21)	0.4
≥ 1 Cerebellar peduncle lesion	26/59 (44)	2/5 (40)	14/27 (52)	10/27 (37)	0.41	26/169 (15)	0/4 (0)	6/39 (15)	20/126 (16)	1
≥ 1 Brainstem lesion	47/62 (76)	4/5 (80)	23/29 (79)	20/28 (71)	0.7	56/191 (29)	3/6 (50)	11/47 (23)	42/138 (30)	0.46
≥ 1 Peri 4th ventricle lesion	26/59 (44)	3/5 (60)	12/27 (44)	11/27 (41)	1	21/169 (12)	0/4 (0)	6/39 (15)	15/126 (12)	0.59
≥ 1 Periventricular lesion	34/62 (55)	2/5 (40)	20/29 (69)	12/28 (43)	0.086	64/191 (34)	2/6 (33)	10/47 (21)	52/138 (38)	0.049
≥ 3 Periventricular lesion	22/61 (36)	2/5 (40)	14/28 (50)	6/28 (21)	0.051	37/186 (20)	1/5 (20)	5/46 (11)	31/135 (23)	0.089
≥ 1 Lesion perpendicular to major axis of corpus callosum	6/62 (10)	1/5 (20)	2/29 (7)	3/28 (11)	0.67	47/191 (25)	1/6 (17)	5/47 (11)	41/138 (30)	0.01
≥ 1 Basal ganglia lesion	27/62 (44)	3/5 (60)	15/29 (52)	9/28 (32)	0.22	10/191 (5)	1/6 (17)	2/47 (4)	7/138 (5)	1

© 2019 Waters P et al. *JAMA Neurology.*

	ADEM					NON-ADEM				
	**All**	**Borderline**	**Positive**	**Negative**	**p value**	**All**	**Borderline**	**Positive**	**Negative**	**p value**
≥ 1 Thalamic lesion	36/62 (58)	3/5 (60)	22/29 (76)	11/28 (39)	0.011	21/191 (11)	1/6 (17)	8/47 (17)	12/138 (9)	0.17
≥ 1 Juxtacortical lesion	49/62 (79)	4/5 (80)	27/29 (93)	18/28 (64)	0.0099	67/191 (35)	4/6 (67)	15/47 (32)	48/138 (35)	0.86
≥ 1 T1 hypointense lesion	12/61 (20)	0/5 (0)	8/29 (28)	4/27 (15)	0.33	65/190 (34)	1/6 (17)	7/47 (15)	57/137 (42)	0.00073
≥ 1 Lesion enhancement	7/49 (14)	1/4 (25)	4/23 (17)	2/22 (9)	0.67	40/181 (22)	1/5 (20)	1/44 (2)	38/132(29)	<0.0001
≥ 1 Gad-negative T1 hypointense leson	12/49 (24)	0/4 (0)	7/23 (30)	5/22 (23)	0.8	43/181 (24)	0/5 (0)	5/44 (11)	38/132(29)	0.025
≥ 1 Presence of discrete lesions	25/62 (40)	1/5 (20)	11/29 (38)	13/28 (46)	0.7	92/191 (48)	1/6 (17)	16/47 (34)	75/138 (54)	0.018
Only well-defined lesions	11/62 (18)	0/5 (0)	4/29 (14)	7/28 (25)	0.33	72/191 (38)	1/6 (17)	10/47 (21)	61/138 (44)	0.0055
Diffuse bilateral pattern	42/62 (68)	5/5 (100)	22/29 (76)	15/28 (54)	0.14	19/191 (10)	1/6 (17)	9/47 (19)	9/138 (7)	0.02
OCBs	7/36 (19)	0/3 (0)	3/17 (18)	4/16 (25)	0.69	40/133 (30)	0/4 (0)	5/32 (16)	35/97 (36)	0.046
MRI evolution										
Years of MRI follow-up	2.03 (1.01-5.03)	1.86 (1.14-4.05)	2.47 (0.70-5.06)	2.00 (1.02-5.00)	0.44	4.01 (1.06-6.01)	5.48 (5.04-6.84)	4.49 (1.13-6.17)	3.95 (1.02-5.99)	0.15
New T2 MRI lesions	9 (15)	0	6 (22)	4 (14)	0.73	63 (33)	1 (17)	9 (18)	53 (39)	0.015
Complete lesion resolution	29/61 (48)	4/5 (80)	15/29 (52)	10/27 (37)	0.3	24/109 (22)	1/3 (33)	11/22 (50)	12/84 (14)	0.00083
Total T2 lesion vol. at last scan	0.00 (0.00-0.37)	0.00 (0.00-0.00)	0.01 (0.00-0.18)	0.00 (0.00-0.44)	0.47	0.04 (0.00-2.64)	0.37 (0.19-0.39)	0.00 (0.00-0.01)	0.31 (0.00-4.06)	0.0039
Total T1 lesion vol. at last scan	0.00 (0.00-0.00)	0.00 (0.00-0.00)	0.00 (0.00-0.00)	0.00 (0.00-0.00)	0.25	0.00 (0.00-0.47)	0.01 (0.00-0.01)	0.00 (0.00-0.00)	0.00 (0.00-1.33)	0.015

Values are reported as median (Interquartile range (IQR)), n(%) or n/N(%). P values are computed between MOG+ and MOG-.
[a]The frequencies of all MRI features pertaining to lesion aspect and location were computed only among patients with brain lesions at baseline. The denominator (N) for each feature corresponds to the number of subjects in which that feature was evaluated.
Analyses of lesion enhancement and Gad-negative T1 hypointensities were further restricted to subjects who had gadolinium administered.
ADEM: Acute Disseminated Encephalomyelitis; ON: Optic Neuritis; TM: Transverse Myelitis; OCBs: Oligoclonal Bands.

© 2019 Waters P et al. *JAMA Neurology.*

eTable 2. Baseline clinical and MRI features of persistently seropositive patients, patients converting to seronegative status, or patients with fluctuating serological status

	**Persistent MOG+**	**Converted to MOG-**	**MOG+ fluctuating**	**p value (persist. MOG+ vs conv. to MOG-)**
Participants	24	38	5	
Years from first to last sample procurement (median (IQR))	4.30 (3.00-5.96)	4.02 (3.01-6.17)	6.97 (4.08-7.44)	0.4
Sex (Female) n(%)	14 (58)	19 (50)	4 (80)	0.7
Age at onset (median (IQR))	9.06 (6.60-13.36)	6.95 (5.28-9.96)	8.97 (8.93-9.67)	0.029
Presenting phenotype n/N(%)				
ADEM	5/24 (21)	17/38 (45)	0/5 (0)	0.1
ADEM with ON	1/24 (4)	1/38 (3)	0/5 (0)	1
ADEM with TM	1/24 (4)	3/38 (8)	0/5 (0)	1
ADEM with ON+TM	0/24 (0)	1/38 (3)	0/5 (0)	1
Monofocal ON	14/24 (58)	9/38 (24)	4/5 (80)	0.013
Polyfocal ON	0/24 (0)	1/38 (3)	0/5 (0)	1
Monofocal TM	2/24 (8)	4/38 (11)	0/5 (0)	1
Polyfocal TM	2/24 (8)	1/38 (3)	1/5 (20)	0.52
ON + TM	0/24 (0)	4/38 (11)	0/5 (0)	0.15
Other	1/24 (4)	2/38 (5)	0/5 (0)	1
Clinical relapses n(%)	9/24 (38)	6/38 (16)	2/5 (40)	0.055
MRI and laboratory baseline features [a]				
Lesions present n/N(%)	9/20 (45)	29/36 (81)	2/5 (40)	0.015
Lesions count (median (IQR))	0 (0-6.5)	13 (1->15)	0 (0-6)	0.0028
≥ 1 Cerebellar lesions n/N(%)	2/9 (22)	12/29 (41)	1/2 (50)	0.44
≥ 1 Cerebellar peduncle lesions n/N(%)	4/9 (44)	11/29 (38)	0/2 (0)	1
≥ 1 Brainstem lesions n/N(%)	4/9 (44)	23/29 (79)	0/2 (0)	0.088
≥ 1 Peri 4th ventricle lesions n/N(%)	4/9 (44)	11/29 (38)	0/2 (0)	1
≥ 1 Periventricular lesions n/N(%)	5/9 (56)	16/29 (55)	2/2 (100)	1
≥ 1 Lesion perpendicular to the major axis of the corpus callosum n/N(%)	2/9 (22)	5/29 (17)	0/2 (0)	1
≥ 1 Basal ganglia lesions n/N(%)	1/9 (11)	8/29 (28)	0/2 (0)	0.41
≥ 1 Thalamic lesions n/N(%)	6/9 (67)	15/29 (52)	0/2 (0)	0.48
≥ 1 Juxtacortical lesions n/N(%)	6/9 (67)	24/29 (83)	2/2 (100)	0.36
≥ 1 T1 hypointense lesions n/N(%)	3/9 (33)	9/29 (31)	2/2 (100)	1
≥ 1 Lesion enhancement n/N(%)	0/5 (0)	4/25 (16)	0/2 (0)	1
≥ 1 Gad-negative T1 hypointense lesions n/N(%)	2/5 (40)	6/25 (24)	2/2 (100)	0.59
≥ 1 Presence of discrete lesions n/N(%)	6/9 (67)	15/29 (52)	2/2 (100)	0.48
Only presence of well-defined lesions n/N(%)	4/9 (44)	9/29 (31)	0/2 (0)	0.69
Diffuse bilateral pattern n/N(%)	3/9 (33)	19/29 (66)	1/2 (50)	0.13
OCBs n/N(%)	2/17 (12)	3/18 (17)	1/3 (33)	1

© 2019 Waters P et al. *JAMA Neurology.*

Baseline anti-MOG titre (IgG H+L assay) (median (IQR))	1200 (800-1600)	800 (400 -1600)	1200 (800-1600)	0.16

P values are computed between persistently MOG+ and converted to MOG-.
[a]The frequencies of all features pertaining to lesion aspect and location were computed only among patients with brain lesions at baseline. The denominator (N) for each feature corresponds to the number of subjects in which that feature was evaluated. Analyses of lesion enhancement and Gad-negative T1 hypointense lesions were further restricted to subjects who had gadolinium administered.
ADEM: Acute Disseminated Encephalomyelitis; ON: Optic Neuritis; TM: Transverse Myelitis; OCBs: Oligoclonal Bands.

© 2019 Waters P et al. *JAMA Neurology.*

eTable 3. Ranking of baseline clinical, MRI, and laboratory features most strongly associated with relapses in seropositive children

Features[a]	Monophasic (n=66)	Relapsing (n=16)	p value	Importance[b]	Ranking
Age at clinical onset	7.03 (4.68-9.87)	8.77(6.72-11.48)	0.098	0.27	1
≥ 1 T1 hypointense lesions n/N(%)	9/61 (15)	5/13 (38)	0.062	0.093	2
Lesion count (median (IQR))	10 (0- >15)	1 (0-6)	0.1	0.07	3
≥ 1 Periventricular lesions n/N(%)	24/61 (39)	5/13 (38)	1	0.064	4
OCBs n/N(%)	5/35 (14)	2/12 (17)	1	0.055	5
≥ 1 Lesion perpendicular to major axis corpus callosum n/N(%)	6/61 (10)	1/13 (8)	1	0.057	6
≥ 1 Enhancing lesion n/N(%)	5/56 (9)	0/10 (0)	1	0.039	7
≥ 1 Gad-negative T1 hypointense lesions n/N(%)	7/56 (12)	5/10 (50)	0.013	0.041	8
ADEM n/N(%)	28/66 (42)	3/16 (19)	0.093	0.036	9
Sex (Female) n/N(%)	35/66 (53)	10/16(62)	0.69	0.033	10
Only well-defined lesions n/N(%)	10/61 (16)	3/13 (23)	0.69	0.027	11
≥ 1 Thalamic lesions n/N(%)	26/61 (43)	3/13 (23)	0.23	0.023	12
ON/TM n/N(%)	37/66 (56)	12/16 (75)	0.26	0.025	13
≥3 Periventricular lesions n/N(%)	17/59 (29)	2/13 (15)	0.49	0.025	14
≥ 1 Brainstem lesions n/N(%)	29/61 (48)	3/13 (23)	0.13	0.02	15
Presence of discrete lesions n/N(%)	21/61 (34)	5/13 (38)	0.76	0.022	16
≥ 1 Juxtacortical lesions n/N(%)	35/61 (57)	6/13 (46)	0.55	0.021	17
≥ 1 Basal ganglia lesions n/N(%)	16/61 (26)	0/13 (0)	0.058	0.019	18
≥ 1 Cerebellar lesions n/N(%)	18/61 (30)	2/13 (15)	0.49	0.014	19
≥ 1 Peri 4th ventricle lesions n/N(%)	16/52 (31)	1/12 (8)	0.16	0.014	20
≥ 1 Cerebellar peduncle lesions n/N(%)	18/53 (34)	0/11 (0)	0.026	0.014	21
Diffuse bilateral pattern n/N(%)	27/61 (44)	3/13 (23)	0.22	0.011	22
Brain lesions present n/N(%)	42/61 (69)	7/13 (54)	0.41	0.0023	23

[a]The frequencies of all MRI features pertaining to lesion aspect and location were computed only among patients with brain lesions at baseline. The denominator (N) for each feature corresponds to the number of subjects in which that feature was evaluated. Analyses of lesion enhancement and Gad-negative T1-hypointensities were further restricted to participants who had gadolinium administered.
[b]*Importance* indicates the GINI impurity-derived importance estimated by the random forest analysis prior to backwards elimination. *Ranking* is the order of importance after the backwards elimination (rank 1 is last eliminated).

© 2019 Waters P et al. *JAMA Neurology.*

eFigure 1. Sensitivity analysis for evolution of serological status

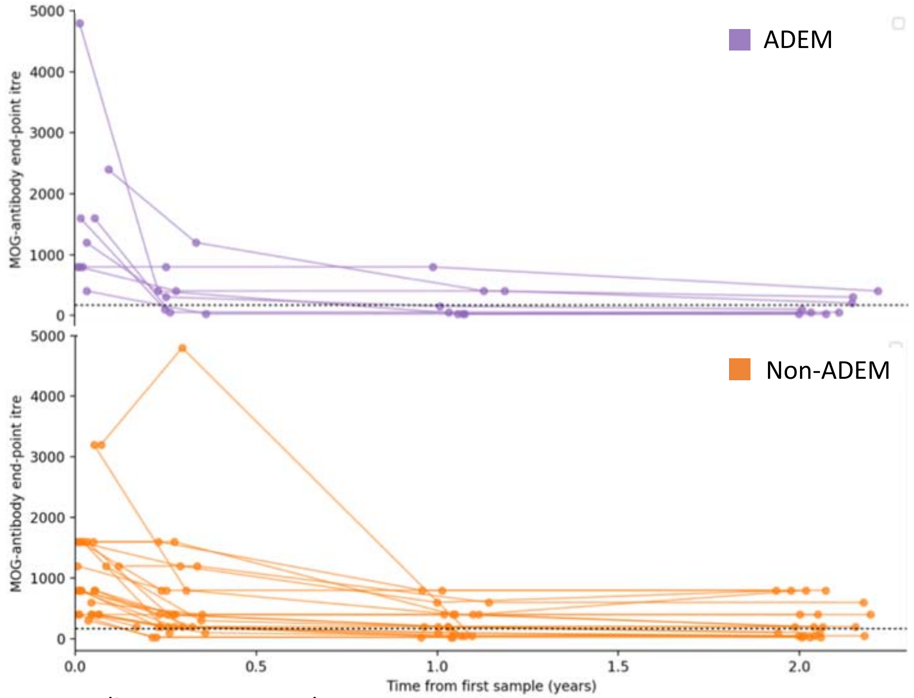

	Baseline	3 Months	1 Year	2 Years
MOG+/All	34/126 (27%)	21/126 (17%)	13/126 (10%)	12/126 (10%)
ADEM MOG+/All ADEM	9/26 (35%)	5/26 (19%)	3/26 (12%)	3/26 (12%)

Analysis of the 126 participants for whom serial samples were available at all timepoints (initial presentation, 3 months, 1 and 2 years). This sub-cohort was similar in all demographic and clinical features to the full study population, with the only exception being a slightly older age at presentation (median 11.75 (IQR 8.03-14.00) in the sub-cohort vs 10.79 (IQR 6.18-13.87) years in the full study cohort, p = 0.045). At three months of follow-up, 13/34 (38%) of participants in the sub-cohort who were MOG+ at presentation became seronegative. At one year, the proportion that became seronegative increased to 21/34 (62%), remaining largely stable at two years (22/34, 65%). When considering only the MOG+ participants presenting with ADEM, 56% had already become seronegative at the earliest timepoint of 3 months.

© 2019 Waters P et al. *JAMA Neurology.*

eFigure 2. Kaplan-Meier curve for risk of a second clinical attack in participants persistently seropositive vs those who converted to seronegative status

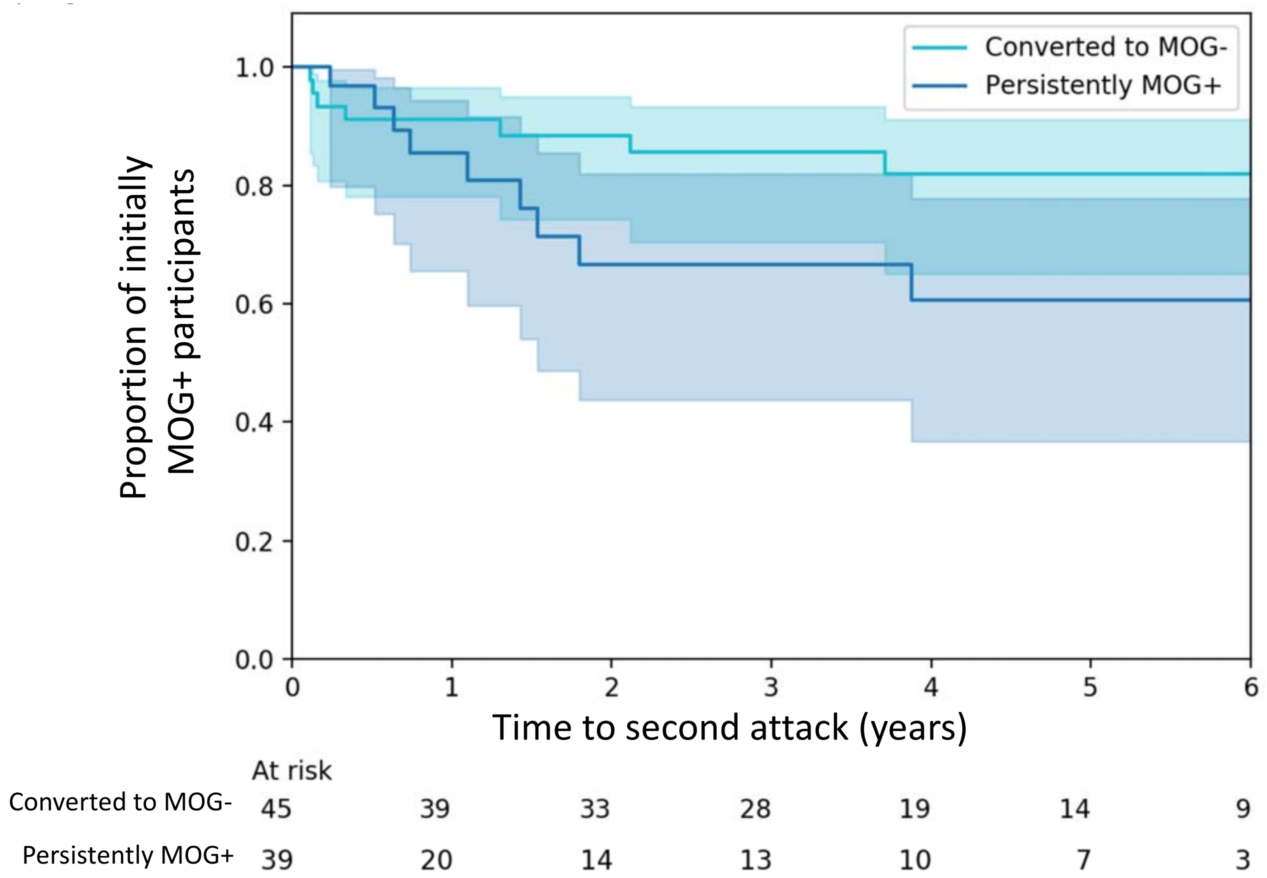

Time to second clinical attack in participants persistently MOG+ vs converted to MOG-. Overall, the likelihood of a relapsing course was low in all participants initially MOG+, but was slightly greater among participants with persistently MOG+ serostatus. In particular, a second clinical attack occurred in 16/84 (19%) initially MOG+ patients, and 9/16 (56%) were persistently positive throughout follow up. One patient had a second clinical attack before converting to persistently MOG-. Of the remaining 6/16 patients who experienced clinical episodes after the detection of the first seronegative sample, 4 persisted seronegative in all serial samples, while 2 had re-appearance of anti-MOG antibodies in subsequent samples.

© 2019 Waters P et al. *JAMA Neurology.*
